# Association between serum carotenoids and hyperuricemia: a cross-sectional study based on NHANES 2001–2006

**DOI:** 10.3389/fnut.2024.1476031

**Published:** 2024-11-07

**Authors:** Pengfei Chen, Lina Miao, Lixiao Zhang, Jianpeng Du, Ming Guo, Dazhuo Shi

**Affiliations:** ^1^Xiyuan Hospital, China Academy of Chinese Medical Sciences, Beijing, China; ^2^Cardiovascular Diseases Center, Xiyuan Hospital, China Academy of Chinese Medical Sciences, Beijing, China

**Keywords:** serum carotenoid, uric acid, hyperuricemia, NHANES, association

## Abstract

**Purpose:**

This study aims to investigate the association between serum carotenoids and hyperuricemia.

**Methods:**

Data were sourced from the National Health and Nutrition Examination Survey (NHANES) between 2001 and 2006. Hyperuricemia was defined as serum uric acid (UA) levels of ≥7 mg/dL for males and ≥ 6 mg/dL for females. Serum carotenoid levels were measured using high-performance liquid chromatography. Multivariate linear regression was used to analyze the correlation between serum carotenoids and UA levels. Multivariate logistic regression and restricted cubic spline (RCS) analyses were performed to explore the potential association between serum carotenoids and hyperuricemia. Additionally, subgroup and interaction analyses were conducted to determine variations across different population groups.

**Result:**

This cross-sectional study included 13,561 participants. Multivariate linear regression analysis revealed that higher levels of serum carotenoids were correlated with lower UA levels. Specifically, the beta coefficients (*β*) and 95% confidence intervals (CIs) were as follows: *α*-carotene (−0.23 [−0.31, −0.15]), β-carotene (−0.30 [−0.38, −0.21]), β-cryptoxanthin (−0.17 [−0.25, −0.09]), lutein/zeaxanthin (−0.12 [−0.20, −0.04]), and total serum carotenoids (−0.25 [−0.33,-0.16]). However, lycopene showed no significant correlation with UA (−0.01 [−0.09, 0.08]). Multivariate logistic regression analysis indicates a significant inverse association between serum carotenoids and the risk of hyperuricemia. The odds ratios (ORs) and 95%CIs were as follows: *α*-carotene (0.61 [0.49, 0.77]), *β*-carotene (0.67 [0.51, 0.86]), β-cryptoxanthin (0.69 [0.51, 0.88]), lutein/zeaxanthin (0.72 [0.56, 0.97]), lycopene (0.82 [0.67, 1.00]) and total serum carotenoids (0.73 [0.57, 0.92]). RCS analysis indicated a potential nonlinear relationship between lycopene and hyperuricemia, with an inflection point at 33.45 μg/dL. Subgroup and interaction analyses demonstrated that the inverse association remained consistent across various demographic groups.

**Conclusion:**

This study found that higher serum carotenoid levels are associated with lower UA levels and reduced risk of hyperuricemia. Notably, while lycopene was associated with reduced hyperuricemia risk, its effect showed some heterogeneity.

## 1 Introduction

Hyperuricemia is a metabolic disorder characterized by elevated serum uric acid (UA) levels resulting from purine metabolism disturbances (1). UA is a byproduct produced from the breakdown of purines, and it is typically excreted through the kidneys. However, when UA production is excessive or excretion is impaired, it can lead to hyperuricemia (2). According to the 2020 epidemiological survey, the prevalence of hyperuricemia among different populations worldwide ranges from 2.6 to 36% (3). A study based on the National Health and Nutrition Examination Survey (NHANES) reported the latest prevalence of hyperuricemia in the U.S. for 2015–2016, with the prevalence in men being 20.2% and in women 20.0% (4). Hyperuricemia is associated with multiple risk factors, including a high-purine diet, alcohol consumption, medication use, and obesity (5–8). Moreover, it is considered a precursor to gout and a risk factor for various chronic diseases, including chronic kidney disease (CKD), cardiovascular diseases (CVD), metabolic syndrome, hypertension, and type 2 diabetes (4, 9, 10). Given the changes in lifestyle and dietary patterns in recent years, the incidence of hyperuricemia may continue to rise.

Dietary habits play a crucial role in the development of hyperuricemia, not only by promoting inflammation and oxidative stress but also through the direct effects of certain nutrients, such as high-protein, high-purine, fructose, and fat-rich diets, which contribute to elevated UA levels (11, 12). Given the known dietary influences on UA levels, attention has turned to specific nutrients, such as carotenoids, which may have a beneficial role. Carotenoids, a group of lipid-soluble pigments responsible for the orange, yellow, and red colors in various fruits and vegetables, are known for their potent antioxidant and anti-inflammatory properties (13). These compounds, commonly found in foods such as lettuce, carrots, tomatoes, and oranges (14, 15), include *α*-carotene, *β*-carotene, β-cryptoxanthin, lutein/zeaxanthin, and lycopene, which together account for over 95% of the carotenoids present in the bloodstream (16). Due to their bioactive properties, carotenoids may play a role in mitigating hyperuricemia by reducing oxidative stress and inflammation, but the specific relationship between serum carotenoids and UA levels remains underexplored (13, 17). This study aims to investigate this association in the general U.S. adult population using the NHANES data.

## 2 Materials and methods

### 2.1 Study population

The NHANES, a comprehensive cross-sectional study conducted by the National Center for Health Statistics (NCHS), was approved by the NCHS research ethics review board, with written informed consent obtained from all participants (18, 19). The NHANES dataset, which employs a multistage, stratified sampling design to produce a sample representative of the non-institutionalized U.S. population, is publicly available and can be accessed at http://www.cdc.gov/nchs/nhanes.htm. This study exclusively utilized data from the NHANES database.

**Figure 1:**
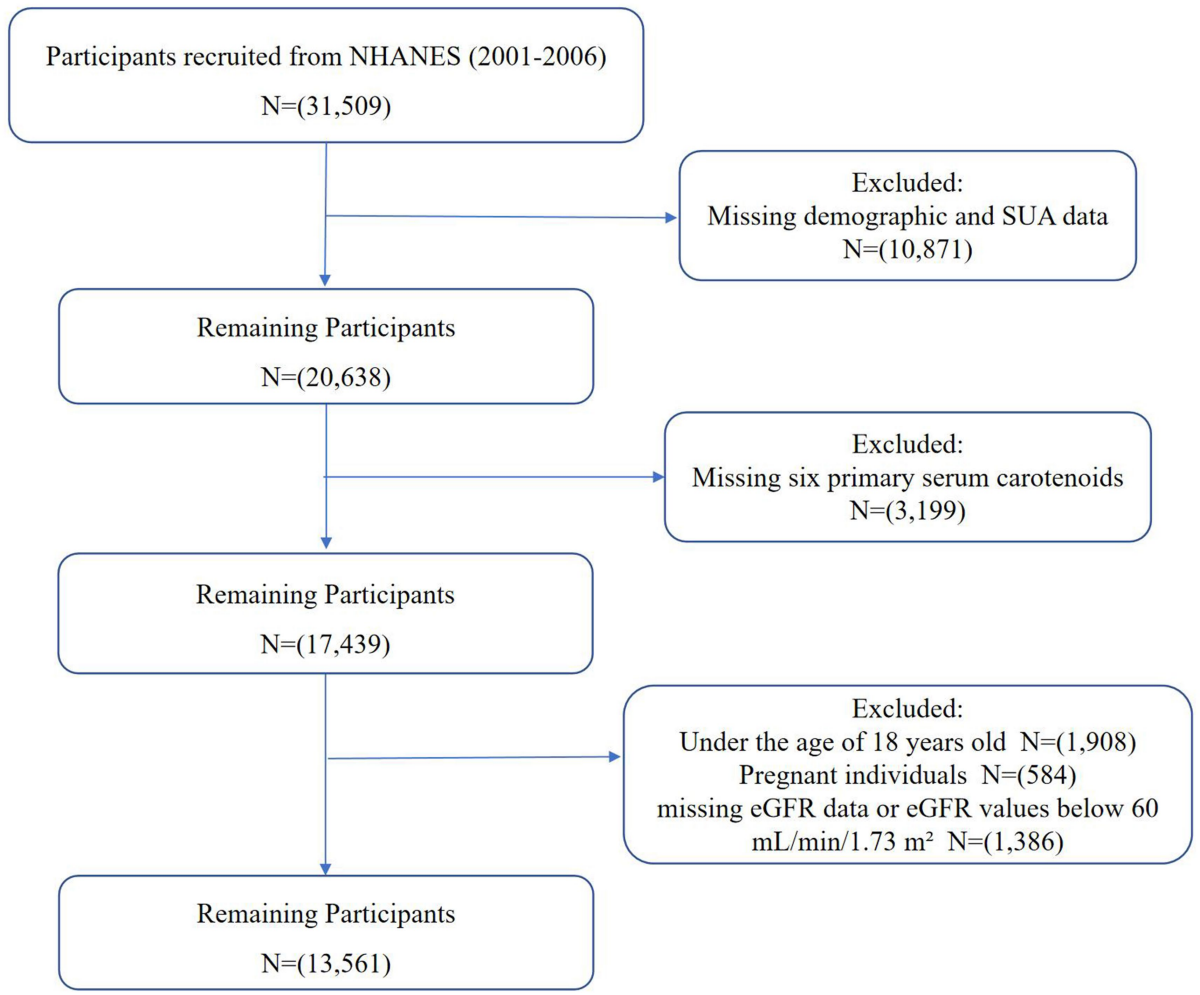
Flowchart of study participants.

This study utilized NHANES data from 2001 to 2006, covering three biennial cycles and initially including 31,509 participants. These years were selected because they provided the most comprehensive and consistent measurements of both serum carotenoids and UA levels, ensuring reliable data for analysis. The exclusion criteria were: (1) participants with missing demographic and UA data (*n* = 10,871), (2) participants with missing data for the six primary serum carotenoids (*n* = 3,199), (3) participants under 18 years old (*n* = 1,908), (4) pregnant individuals (*n* = 584), and (5) participants with missing estimated glomerular filtration rate (eGFR) data or eGFR values below 60 mL/min/1.73 m^2^ (*n* = 1,386). Ultimately, 13,561 participants were included in this study, as shown in Figure 1.

### 2.2 Exposure variable and outcomes

The main variables in this study were the concentrations of UA and six primary serum carotenoids, including *α*-carotene, *β*-carotene, β-cryptoxanthin, lutein/zeaxanthin, and lycopene. Hyperuricemia was defined as UA levels ≥7 mg/dL for males and ≥ 6 mg/dL for females (3). UA levels were measured using either Beckman Synchron LX20 or a Beckman UniCel^®^ DxC800 Synchron, following the oxidation of UA to allantoin and hydrogen peroxide by uricase.

In NHANES 2001–2002 and 2005–2006, serum carotenoids were measured using high-performance liquid chromatography (HPLC). In NHANES 2002–2003, using a similar HPLC method with multiwavelength photodiode-array absorbance detection at 450 nm to analyzed serum carotenoids. Data from NHANES 2003–2004 were adjusted using a regression method to match the carotenoid measurements from the HPLC method. Total serum carotenoid (TSC) levels were calculated by summing the six primary serum carotenoids.

### 2.3 Covariates

Demographic data were collected through questionnaire interviews and included age, sex (male, female), race (Non-Hispanic White, Non-Hispanic Black, Mexican American, other Hispanic, other race), and education status (below high school, high school, above high school). The family poverty income ratio (PIR) was used to categorize participants into different income levels (20, 21). We categorized participants into three groups: low income (PIR <1.0), moderate income (PIR 1.0–3.0), and high income (PIR >3.0). BMI was calculated by dividing weight (in kilograms) by height (in meters squared) and categorized as <25, 25–29, and > 29 kg/m^2^. Alcohol status was classified as >10 drinks/month, 5–10 drinks/month, 1–5 drinks/month, and no drinks. Smoking status was divided into never (<100 cigarettes in lifetime), current (≥100 cigarettes and currently smoking), and former (≥100 cigarettes but not currently smoking). Diabetes was diagnosed based on fasting glucose level (≥7.0 mmol/L), glycohemoglobin (≥6.5%), the use of anti-diabetic medications or insulin, or a prior physician diagnosis of diabetes. Hypertension was diagnosed based on systolic blood pressure ≥ 140 mmHg, diastolic blood pressure ≥ 90 mmHg, a prior diagnosis, or a history of anti-hypertensive medication use. Blood pressure was measured using a clinically validated automated monitor following the NHANES standardized protocol. After 5 min of seated rest, three consecutive readings were taken, with a fourth attempt if needed. The final blood pressure value was the average of the last two valid readings. CVD was self-reported doctor-diagnosed conditions, such as coronary heart disease, heart failure, heart attack, stroke, and angina pectoris. Laboratory covariates included serum creatinine, glucose, eGFR, blood urea nitrogen (BUN), serum total cholesterol (STC), and serum triglycerides (STG). Participants’ medication histories were collected, including the use of urate-lowering therapies (e.g., allopurinol, febuxostat), antihypertensive medications such as thiazide diuretics or angiotensin receptor blockers (ARBs), and loop diuretics used for heart failure treatment.

### 2.4 Statistical analysis

For all statistical analyses, NHANES sampling weights were applied in R 4.3.3 to account for the stratification and complexity of the survey design. The participants in this study were weighted to represent a population of 192,694,927. The categorical variables were displayed as unweighted counts (percentages), and the continuous variables were presented as weighted medians (interquartile range). A two-sided *p* < 0.05 was set as the threshold for statistical significance.

Multivariate linear regression was utilized to estimate beta coefficients (*β*) and 95% confidence intervals (CIs) to explore the associations between serum carotenoids and UA. Serum carotenoids were categorized into four quartiles, with the lowest quartile serving as the reference group. Trend tests (p-trend) across quartiles were conducted using the median serum carotenoid levels in each quartile as a linear variable. Additionally, natural ln-transformed serum carotenoids were analyzed as continuous variables in the linear regression. In Model 1, each serum carotenoid was the sole independent variable. Model 2 included adjustments for sex, race, age, poverty status, smoking status, alcohol status, education, and BMI. Model 3 built on Model 2 by further adjusting for diabetes, hypertension, medication history, CVD and serum creatinine.

Multivariate logistic regression was used to estimate prevalence odds ratios (ORs) and 95%CIs to examine the associations between serum carotenoids and hyperuricemia. Serum carotenoid quartiles were treated as categorical variables, while ln-transformed serum carotenoids were used as continuous variables. Additionally, restricted cubic splines (RCS) were applied to investigate potential non-linear relationships. Wald tests for the RCS coefficients were used to calculate *p*-values for non-linear trends. Subgroup heterogeneity was assessed through interaction analyses.

## 3 Results

### 3.1 Baseline characteristics

Table 1 presents the weighted baseline characteristics for participants with and without hyperuricemia. The study included 13,561 participants from the NHANES 2001–2006 survey, comprising 6,519 (48.07%) males and 7,042 (51.93%) females, with an average age of 46.17 ± 16.83 years. Among them, 2,614 (19.28%) were diagnosed with hyperuricemia. The majority of participants were Non-Hispanic White (52.29%), 16.89% lived in poverty, and 70.94% had a high school diploma or higher. Additionally, 51.21% had never smoked, and 29.16% had never consumed alcohol. Diabetes was diagnosed in 13.21% of the participants, hypertension in 31.84% and CVD in 11.57%. The median (interquartile range) serum concentrations were 2.7 (1.4, 5.1) μg/dL for *α*-carotene, 13 (7, 23) μg/dL for *β*-carotene, 9 (5, 11) μg/dL for β-cryptoxanthin, 14 (10, 20) μg/dL for lutein/zeaxanthin, 22 (15, 30) μg/dL for lycopene, and 64 (47, 86) μg/dL for TSC. Participants with hyperuricemia exhibited lower levels of the six primary serum carotenoids and eGFR, alongside elevated concentrations of creatinine, blood glucose, BUN, STC, STG, as well as higher systolic and diastolic blood pressure, compared to those without hyperuricemia.

**Table 1:** Baseline characteristics of study participants stratified by hyperuricemia status.

Characteristic	Overall, *N* = 13,561	Non-hyperuricemia, *N* = 10,947	Hyperuricemia, *N* = 2,614	*p*-value
Age (years)	46.17 (16.83)	45.14 (16.39)	50.55 (17.93)	<0.001
Gender, *n* (%)				<0.001
Female	7,042 (51.93)	5,921 (54.09)	1,121 (42.88)	
Male	6,519 (48.07)	5,026 (45.91)	1,493 (57.12)	
Race, *n* (%)				<0.001
Non-Hispanic White	7,091 (52.29)	5,640 (51.55)	1,451 (55.52)	
Non-Hispanic Black	2,683 (19.78)	2,044 (18.68)	639 (24.44)	
Mexican American	2,796 (20.62)	2,455 (22.43)	341 (13.04)	
Other Hispanic	475 (3.50)	399 (3.65)	76 (2.91)	
Other Race/MultiRacial	516 (3.81)	409 (3.74)	107 (4.09)	
Education, *n* (%)				<0.001
Below high school	3,941 (29.06)	3, 177 (29.03)	764 (29.23)	
High school	3,263 (24.06)	2,562 (23.41)	701 (26.81)	
Above high school	6,357 (46.88)	5,208 (47.58)	1,149 (43.96)	
Alcohol consumption, *n* (%)				<0.001
1–5 drinks/mouths	8,724 (64.33)	7,123 (65.08)	1,601 (61.23)	
5–10 drinks/mouths	295 (2.18)	220 (2.01)	75 (2.87)	
>10 drinks/mouths	587 (4.33)	429 (3.92)	158 (6.04)	
Non drinks	3,955 (29.16)	3,175 (29.00)	780 (29.84)	
Smoke, *n* (%)				<0.001
Never	6,944 (51.21)	5,704 (52.12)	1,240 (47.44)	
Current	3,035 (22.38)	2,522 (23.04)	513 (19.62)	
Former	3,582 (26.41)	2,721 (24.86)	861 (32.93)	
Poverty-income ratio, *n* (%)				<0.001
<1.0	2,290 (16.89)	1,900 (17.36)	390 (14.92)	
1.1–3.0	6,114 (45.08)	4,861 (44.41)	1,253 (47.94)	
>3.0	5,157 (38.03)	4,186 (38.24)	971 (37.13)	
BMI, kg/m2 (IQR)	28 (24, 31)	28 (23, 31)	31 (27, 35)	<0.001
Diabetes, *n* (%)	1,791 (13.21)	1,283 (11.72)	508 (19.43)	<0.001
Hypertension, *n* (%)	4,320 (31.84)	2,948 (26.94)	1,372 (52.49)	<0.001
CVD, *n* (%)	1,569 (11.57)	1,020 (9.32)	549 (21)	<0.001
Mean serum carotenoids, ug/dL (IQR)	10.6 (7.8, 14.4)	10.9 (8.0, 14.8)	9.4 (6.9, 12.9)	<0.001
Total serum carotenoids, ug/dL (IQR)	64 (47, 86)	65 (48, 89)	56 (41, 78)	<0.001
α-carotene, ug/dL (IQR)	2.7 (1.4, 5.1)	2.8 (1.5, 5.5)	2.1 (1.2, 4.0)	<0.001
β-carotene, ug/dL (IQR)	13 (7, 23)	13 (8, 24)	10 (6, 18)	<0.001
β-cryptoxanthin, ug/dL (IQR)	9 (5, 11)	10 (5, 12)	8 (4, 10)	<0.001
Lutein and zeaxanthin, ug/dL (IQR)	14 (10, 20)	14 (10, 20)	13 (9, 19)	<0.001
Lycopene, ug/dL (IQR)	22 (15, 30)	22 (15, 30)	21 (14, 29)	<0.001
Cholesterol, mg/dL (IQR)	197 (173, 226)	196 (172, 224)	203 (177, 234)	<0.001
Triglycerides, mg/dL (IQR)	114 (77, 175)	106 (73, 163)	152 (105, 220)	<0.001
Creatinine, mg/dL(IQR)	0.90 (0.80, 1.00)	0.90 (0.70, 1.00)	1.00 (0.90, 1.10)	<0.001
Blood urea nitrogen, mg/dL(IQR)	12 (9, 16)	12 (9, 15)	15 (11, 19)	<0.001
Glucose, mmol/L(IQR)	5.38(5, 5.91)	5.33(4.94, 5.83)	5.64 (5.21, 6.29)	<0.001
eGFR, mL/min/1.73 m^2^(IQR)	90.24 (73.23, 106.47)	92.65 (77.32, 109.27)	75.39 (55.26, 93.16)	<0.001
Systolic blood pressure, mmHg(IQR)	120 (110, 134)	118 (110, 132)	126 (114, 138)	<0.001
Diastolic blood pressure, mmHg(IQR)	70 (62, 78)	70 (62, 78)	72 (62, 80)	<0.001

Normally distributed continuous variables are described as means ± SEs, and continuous variables without a normal distribution are described as medians (interquartile ranges). Categorical variables are presented as numbers (percentages). All estimates accounted for complex survey designs.

The study participants were divided into four groups based on TSC quartiles, as shown in Table 2. Significant differences were observed across all TSC quartiles for age, race, sex, smoking status, poverty status, education, diabetes, hypertension, glucose, BMI, STC, STG, BUN, eGFR, blood pressure, and serum carotenoids concentrations. However, no significant differences were found for alcohol status, medication histories and creatinine levels (*p* > 0.05).

**Table 2:** Baseline characteristics of study participants stratified by TSC.

Characteristic	Overall, *N* = 13,561	Q1, *N* = 3,294 (<47 μg/dL)	Q2, *N* = 3,269 (47–64 μg/dL)	Q3, *N* = 3,356 (64-86 μg/dL)	Q4, *N* = 3,642 (>86 μg/dL)	*p*-value
Age (years)	46.17 (16.83)	45.76 (16.84)	44.73 (16.94)	44.73 (16.94)	49.20 (16.53)	<0.001
Gender, *n* (%)						<0.001
Female	7,042 (51.93)	1,564 (47.48)	1,649 (50.45)	1,712 (51.01)	2,117 (58.13)	
Male	6,519 (48.07)	1,730 (52.52)	1,620 (49.55)	1,644 (48.99)	1,525 (41.87)	
Race, *n* (%)						0.011
Non-Hispanic White	7,091 (52.29)	1,855 (56.31)	1,723 (52.7)	1,693 (50.44)	1,820 (49.98)	
Non-Hispanic Black	2,683 (19.78)	697 (21.16)	684 (20.93)	654 (19.49)	648 (17.79)	
Mexican American	2,796 (20.62)	541 (16.42)	626 (19.15)	754 (22.46)	875 (24.03)	
Other Hispanic	475 (3.50)	102 (3.10)	122 (3.73)	123 (3.67)	128 (3.51)	
Other Race/MultiRacial	516 (3.81)	99 (3.00)	114 (3.49)	132 (3.93)	171 (4.69)	
Education, *n* (%)						<0.001
Below high school	3,941 (29.06)	1,124(34. 13)	961 (29.40)	925 (27.56)	931 (25.56)	
High school	3,263 (24.06)	914 (27.75)	836 (25.57)	807 (24.05)	706 (19.38)	
Above high school	6,357 (46.88)	1,256 (38.12)	1,472 (45.04)	1,624 (48.39)	2,005 (55.06)	
Alcohol consumption, *n* (%)						0.6
1–5 drinks/mouths	8,724 (64.33)	2,146 (65.15)	2,110 (64.55)	2,190 (65.26)	2,278 (62.55)	
5–10 drinks/mouths	295 (2.18)	76 (2.31)	66 (2.02)	79 (2.35)	74 (2.03)	
>10 drinks/mouths	587 (4.33)	161 (4.89)	147 (4.5)	114 (3.40)	165 (4.53)	
Non drinks	3,955 (29.16)	911 (27.65)	946 (28.93)	973 (28.99)	1, 125 (30.89)	
Smoke, *n* (%)						<0.001
Never	6,944 (51.21)	1,273 (38.64)	1,567 (47.94)	1,835 (54.68)	2,269 (62.29)	
Current	3,035 (22.38)	1,186 (36.00)	859 (25.80)	644 (19.19)	346 (9.50)	
Former	3,582 (26.41)	835 (25.35)	843 (26.26)	877 (26.13)	1,027 (47.21)	
Poverty-income ratio, n (%)						<0.001
<1.0	2,290 (16.89)	674 (20.46)	589 (18.02)	539 (16.06)	488 (13.40)	
1.1–3.0	6,114 (45.08)	1,670 (50.70)	1,501 (45.91)	1,478 (44.04)	1,465 (40.22)	
>3.0	5,157 (38.03)	950 (28.84)	1,179 (36.08)	1,339 (39.89)	1,689 (46.38)	
BMI, kg/m2 (IQR)	28 (24, 31)	30 (25, 34)	29 (24, 33)	28 (24, 31)	26 (23, 29)	<0.001
Diabetes, *n* (%)	1,791 (13.21)	607 (18.43)	452 (13.83)	362 (10.79)	370 (10. 16)	<0.001
Hypertension, *n* (%)	4,320 (31.84)	1,244 (37.77)	1,040 (31.82)	979 (29. 18)	1,057 (29.02)	<0.001
CVD, *n* (%)	1,569 (11.57)	548 (16.67)	397 (12.14)	333 (9.92)	291 (7.99)	<0.001
Medication histories, n (%)	811 (5.98)	202 (6.13)	208 (6.36)	198 (5.90)	203 (5.57)	0.40
Mean serum carotenoids, ug/dL(IQR)	10.6 (7.8, 14.4)	6. 1 (5.0, 7.0)	9.2 (8.5, 9.9)	12.3 (11.4, 13.3)	18.1 (15.9, 22.2)	<0.001
Total serum carotenoids, ug/dL (IQR)	64 (47, 86)	37 (30, 42)	55 (51, 59)	74 (68, 80)	108 (95, 133)	<0.001
α-carotene, ug/dL (IQR)	2.7 (1.4, 5. 1)	1.2 (0.7, 1.8)	2.0 (1.3, 3.3)	3.4 (2.1, 5.1)	6.9 (4.3,11.3)	<0.001
β-carotene, ug/dL (IQR)	13 (7, 23)	6 (4, 8)	10 (7, 14)	16 (11, 22)	33 (23, 50)	<0.001
β-cryptoxanthin, ug/dL (IQR)	9 (5, 11)	4 (3, 5)	7 (5, 8)	10 (6, 12)	16 (9, 20)	<0.001
Lutein and zeaxanthin, ug/dL (IQR)	14 (10, 20)	9 (7, 11)	13 (10, 16)	16 (13, 20)	22 (18, 29)	<0.001
Lycopene, ug/dL (IQR)	22 (15, 30)	14 (9, 18)	22 (16, 27)	26 (20, 33)	29 (22, 38)	<0.001
Cholesterol, mg/dL (IQR)	197 (173, 226)	181 (157, 206)	194 (170, 220)	201 (179, 231)	215 (190, 242)	<0.001
Triglycerides, mg/dL (IQR)	114 (77, 175)	124 (81, 182)	118 (78, 179)	112 (77, 174)	104 (71, 163)	<0.001
Creatinine, mg/dL (IQR)	0.90 (0.80, 1.00)	0.90 (0.80,1.00)	0.90 (0.80,1.00)	0.90 (0.80, 1.00)	0.90 (0.70, 1.00)	0.5
Blood urea nitrogen, mg/dL(IQR)	12 (9, 16)	12 (9, 15)	12 (9, 15)	12 (10, 16)	13 (10, 16)	<0.001
Glucose, mg/dL(IQR)	5.38(5.00, 5.91)	5.50(5.07, 6.22)	5.38(5.00, 5.95)	5.34(4.98, 5.80)	5.30(4.89, 5.77)	<0.001
eGFR, mL/min/1.73 m^2^(IQR)	90.24 (73.23, 106.47)	89.31 (71.77, 104.25)	90.58 (74.12, 106.47)	92.51 (75.78, 108.89)	88.53 (71.34, 107.21)	<0.001
Systolic blood pressure, mmHg(IQR)	120 (110, 134)	122 (112, 136)	120 (110, 134)	110 (118, 132)	120 (110, 132)	<0.001
Diastolic blood pressure, mmHg(IQR)	70 (62, 78)	70 (62, 80)	70 (62, 78)	70 (62, 78)	70 (62, 78)	0.02

Normally distributed continuous variables are described as means ± SEs, and continuous variables without a normal distribution are described as medians (interquartile ranges). Categorical variables are presented as numbers (percentages). All estimates accounted for complex survey designs.

### 3.2 Association between serum carotenoids and UA levels

Table 3 shows the results of examining the associations between serum carotenoids and UA levels. In all three models, the highest quartiles of serum carotenoids concentrations were significantly negatively associated with UA levels compared to their respective lowest quartiles. After adjusting for all covariates, the inverse associations remained robust for *α*-carotene (−0.23 [−0.31, −0.15]), *β*-carotene (−0.30 [−0.38, −0.21]), *β*-cryptoxanthin (−0.17 [−0.25, −0.09]), lutein/zeaxanthin (−0.12 [−0.20, −0.04]), and TSC (−0.25 [−0.33, −0.16]). These findings suggest that higher carotenoid levels in the blood may be clinically beneficial by helping to maintain lower UA levels, which could reduce the risk of developing hyperuricemia. However, lycopene showed no significant correlation with UA after adjusting for all covariates, the β and 95%CI was −0.01 [−0.09, 0.08], indicating variability in the effects of different carotenoids. When serum carotenoids were included in the linear regression model as continuous variables, similar results were found: there was a negative correlation between serum carotenoids and UA, while lycopene showed no significant correlation with UA level (Table 4).

**Table 3:** Associations between serum carotenoid levels by quartiles and serum uric acid levels.

Carotenoids	Range	Model 1	Model 2	Model 3
α-Carotene				
Q1	<1.4	1	1	1
Q2	1.4–2.7	−0.18 (−0.26, −0.09)	−0.05 (−0.13, −0.03)	−0.05 (−0.13, 0.03)
Q3	2.7–5.1	−0.31 (−0.39, −0.22)	−0.11 (−0.19, −0.03)	−0.12 (−0.19, −0.04)
Q4	>5. 1	−0.64 (−0.72, −0.56)	−0.24 (−0.32, −0.17)	−0.23 (−0.31, −0.15)
p-trend		<0.01	<0.01	<0.01
β-carotene				
Q1	<7.29	1	1	1
Q2	7.29–12.50	−0.29 (−0.38, −0.21)	−0.13 (−0.21, −0.06)	−0.14 (−0.22, −0.07)
Q3	12.50–22.85	−0.42 (−0.50, −0.34)	−0.20 (−0.28, −0.12)	−0.21 (−0.29, −0.13)
Q4	>22.85	−0.75 (−0.83, −0.67)	−0.31 (−0.39, −0.23)	−0.30 (−0.38, −0.21)
p-trend		<0.01	<0.01	<0.01
β-cryptoxanthin				
Q1	<4.58	1	1	1
Q2	4.58–7.10	−0.16 (−0.24, −0.08)	−0.05 (−0.11, 0.03)	−0.04 (−0.11, 0.04)
Q3	7.10–11.38	−0.21 (−0.29, −0.12)	−0.05 (−0.12, −0.04)	−0.04 (−0.12, −0.03)
Q4	> 11.38	−0.49 (−0.57, −0.41)	−0.18 (−0.26, −0.10)	−0.17(−0.25, −0.09)
p-trend		<0.01	<0.01	<0.01
Lutein and zeaxanthin				
Q1	<10.10	1	1	1
Q2	10.10–14.09	−0.12 (−0.20, −0.03)	−0.08 (−0.15, −0.01)	−0.07 (−0.14, 0.01)
Q3	14.09–19.60	−0.16 (−0.24, −0.08)	−0.09 (−0.16, −0.01)	−0.08 (−0.16, −0.01)
Q4	>19.60	−0.28 (−0.36, −0.20)	−0.14 (−0.21, −0.06)	−0.12 (−0.20, −0.04)
p-trend		<0.01	<0.01	<0.01
Lycopene				
Q1	<15.10	1	1	1
Q2	15.10–22.00	−0.17 (−0.25, −0.08)	−0.06 (−0.14, 0.03)	−0.05 (−0.14, 0.03)
Q3	22.00–29.68	−0.14 (−0.23, −0.06)	−0.04 (−0.12, 0.04)	−0.04 (−0.12, 0.04)
Q4	>29.68	−0.04 (−0.12, 0.04)	−0.01 (−0.09, 0.07)	−0.01 (−0.09, 0.08)
p-trend		0.352	0.428	0.554
Total carotenoids				
Q1	<47.20	1	1	1
Q2	47.20–64.08	−0.16 (−0.25, −0.08)	−0.11 (−0.19, −0.04)	−0.10 (−0.18, −0.02)
Q3	64.08–85.86	−0.27 (−0.36, −0.19)	−0.17 (−0.25, −0.10)	−0.17 (−0.24, −0.09)
Q4	>85.86	−0.54 (−0.62, −0.46)	−0.27 (−0.35, −0.18)	−0.25 (−0.33, −0.16)
p-trend		<0.01	<0.01	<0.01

The serum carotenoids was categorized into four quartiles and tests for trend (p–trend) based on variable containing the median value for each quartiles. ln-transformed serum carotenoids also was utilized as continuous variables and p-value was used to test significance. Model 1 was a crude model with no adjusted covariates; Model 2 was adjusted for sex, age, race, poverty status, education, smoking status, alcohol status, and BMI; Model 3 was further adjusted for diabetes, hypertension, CVD, medication history, and serum creatinine based on Model 2.

**Table 4:** Associations between serum carotenoid levels as continuous variables and serum uric acid levels.

Carotenoids	Model 1	Model 2	Model 3
α-Carotene	−0.25 (−0.28, −0.22), *p* < 0.01	−0.10 (−0.13, −0.07), *p* < 0.05	−0.09 (−0.12, −0.06), *p* < 0.05
β-carotene	−0.34 (−0.37, −0.31), *p* < 0.01	−0.15 (−0.19, −0.12), *p* < 0.01	−0.14 (−0.18, −0.11), *p* < 0.01
β-cryptoxanthin	−0.24 (−0.28, −0.20), *p* < 0.01	−0.09 (−0.12, −0.05), *p* < 0.05	−0.08 (−0.12, −0.04), *p* < 0.05
Lutein and zeaxanthin	−0.22 (−0.28, −0.16), *p* < 0.01	−0.10 (−0.16, −0.04), *p* < 0.05	−0.09 (−0.15, −0.03), *p* < 0.05
Lycopene	−0.03 (−0.08, 0.02), *p* = 0.75	−0.01 (−0.05, 0.06), *p* = 0.80	0.00 (−0.05, 0.06), *p* = 0.90
Total Carotenoids	−0.42 (−0.38, −0.36), *p* < 0.01	−0.20 (−0.26, −0.13), *p* < 0.01	−0.18 (−0.25, −0.12), *p* < 0.01

ln-transformed serum carotenoids also was utilized as continuous variables and p-value was used to test significance. Model 1 is a crude model without any adjusted covariates; Model 2 is adjusted for sex, age, race, poverty status, education, smoking status, alcohol consumption, and BMI; Model 3 further adjusts for diabetes, hypertension, CVD, medication history, and serum creatinine, based on adjustments in Model 2.

### 3.3 Association between serum carotenoids and the risk of hyperuricemia

Table 5 presents the findings of examining the association between serum carotenoid levels and hyperuricemia risk. In all three models, the highest quartiles of all serum carotenoids were significantly negatively associated with the risk of hyperuricemia compared to their respective lowest quartiles. After adjusting for all covariates, the inverse associations remained robust for *α*-carotene (0.61 [0.49, 0.77]), *β*-carotene (0.67 [0.51, 0.86]), β-cryptoxanthin (0.69 [0.51, 0.88]), lutein/zeaxanthin (0.72 [0.56, 0.97]), lycopene (0.82 [0.67, 1.00]) and total serum carotenoids (0.73 [0.57, 0.92]). These results underscore the potential clinical value of maintaining adequate serum carotenoid levels, which may help lower the risk of hyperuricemia, likely due to the antioxidant and anti-inflammatory properties of carotenoids. When serum carotenoids were analyzed as continuous variables in the multivariate logistic regression model, a similar conclusion was reached: there was a negative correlation between serum carotenoid levels and the risk of hyperuricemia. This further supports the robustness of the inverse association across different analytical approaches (Table 6).

**Table 5:** Associations between serum carotenoid levels by quartiles and hyperuricemia.

Carotenoids	Range	Model 1	Model 2	Model 3
α-Carotene				
Q1	<1.4	1	1	1
Q2	1.4–2.7	0.80 (0.71, 0.91)	0.86 (0.73, 1.00)	0.82 (0.67, 1.00)
Q3	2.7–5.1	0.72 (0.62, 0.84)	0.78 (0.65, 0.94)	0.70 (0.57, 0.85)
Q4	>5. 1	0.42 (0.36, 0.49)	0.56 (0.48, 0.67)	0.61 (0.49, 0.77)
P for trend		<0.01	<0.01	<0.01
β-carotene				
Q1	<7.29	1	1	1
Q2	7.29–12.50	0.68 (0.59, 0.79)	0.70 (0.59, 0.83)	0.68 (0.53, 0.86)
Q3	12.50–22.85	0.58 (0.50, 0.67)	0.61 (0.52, 0.71)	0.59 (0.50, 0.71)
Q4	>22.85	0.44 (0.37, 0.51)	0.53 (0.44, 0.64)	0.67 (0.51, 0.86)
P for trend		<0.01	<0.01	0.02
β-cryptoxanthin				
Q1	<4.58	1	1	1
Q2	4.58–7.10	0.82 (0.70, 0.96)	0.93 (0.78, 1. 10)	0.96 (0.78, 1.17)
Q3	7.10–11.38	0.73 (0.63, 0.86)	0.91 (0.77, 1.09)	0.95 (0.73, 1.20)
Q4	> 11.38	0.45 (0.38, 0.53)	0.60 (0.50, 0.72)	0.69 (0.51, 0.88)
P for trend		<0.01	<0.01	<0.01
Lutein and zeaxanthin				
Q1	<10.10	1	1	1
Q2	10.10–14.09	0.76 (0.66, 0.89)	0.80 (0.67, 0.96)	0.75 (0.54, 0.93)
Q3	14.09–19.60	0.69 (0.60, 0.80)	0.74 (0.63, 0.87)	0.74 (0.61, 0.90)
Q4	>19.60	0.61 (0.51, 0.73)	0.70 (0.57, 0.86)	0.72 (0.56, 0.97)
P for trend		<0.01	<0.01	0.15
Lycopene				
Q1	<15.10	1	1	1
Q2	15.10–22.00	0.77 (0.67, 0.89)	0.87 (0.74, 1.02)	0.86 (0.66, 1.12)
Q3	22.00–29.68	0.76 (0.67, 0.87)	0.84 (0.72, 0.98)	0.92 (0.73, 1.19)
Q4	>29.68	0.68 (0.60, 0.78)	0.75 (0.63, 0.89)	0.82 (0.67, 1.00)
P for trend		<0.01	<0.01	0.21
Total Carotenoids				
Q1	<47.20	1	1	1
Q2	47.20–64.08	0.78 (0.70, 0.86)	0.81 (0.73, 0.90)	0.83 (0.68, 0.98)
Q3	64.08–85.86	0.62 (0.54, 0.72)	0.70 (0.61, 0.80)	0.69 (0.55, 0.87)
Q4	>85.86	0.45 (0.38, 0.52)	0.56 (0.47, 0.67)	0.73 (0.57, 0.92)
P for trend		<0.01	<0.01	0.03

The serum carotenoids was categorized into four quartiles and tests for trend (p–trend) based on variable containing the median value for each quartiles. ln-transformed serum carotenoids also was utilized as continuous variables and p-value was used to test significance. Model 1 was a crude model with no adjusted covariates; Model 2 was adjusted for sex, age, race, poverty status, education, smoking status, alcohol status, and BMI; Model 3 was further adjusted for diabetes, hypertension, CVD, medication history, and serum creatinine based on Model 2.

**Table 6:** Associations between serum carotenoid levels as continuous variables and hyperuricemia.

Carotenoids	Model 1	Model 2	Model 3
α-Carotene	0.73 (0.69, 0.77), *p* < 0.01	0.79 (0.74, 0.84), *p* < 0.01	0.80 (0.73, 0.87), *p* < 0.01
β-carotene	0.67 (0.63, 0.72), *p* < 0.01	0.71 (0.66, 0.77), *p* < 0.01	0.75 (0.68, 0.84), *p* < 0.01
β-cryptoxanthin	0.68 (0.63, 0.74), *p* < 0.01	0.77 (0.70, 0.85), *p* < 0.01	0.82 (0.73, 0.93), *p* < 0.01
Lutein and zeaxanthin	0.68 (0.61, 0.76), *p* < 0.01	0.74 (0.65, 0.85), *p* < 0.01	0.77 (0.65, 0.91), *p* < 0.01
Lycopene	0.79 (0.74, 0.85), *p* < 0.05	0.83 (0.76, 0.91), *p* < 0.05	0.84 (0.73, 0.97), *p* < 0.05
Total Carotenoids	0.54 (0.49, 0.59), *p* < 0.01	0.63 (0.56, 0.71), *p* < 0.01	0.68 (0.57, 0.81), *p* < 0.01

ln-transformed serum carotenoids also was utilized as continuous variables and p-value was used to test significance. Model 1 is a crude model without any adjusted covariates; Model 2 is adjusted for sex, age, race, poverty status, education, smoking status, alcohol consumption, and BMI; Model 3 further adjusts for diabetes, hypertension, CVD, medication history, and serum creatinine, based on adjustments in Model 2.

**Figure 2:**
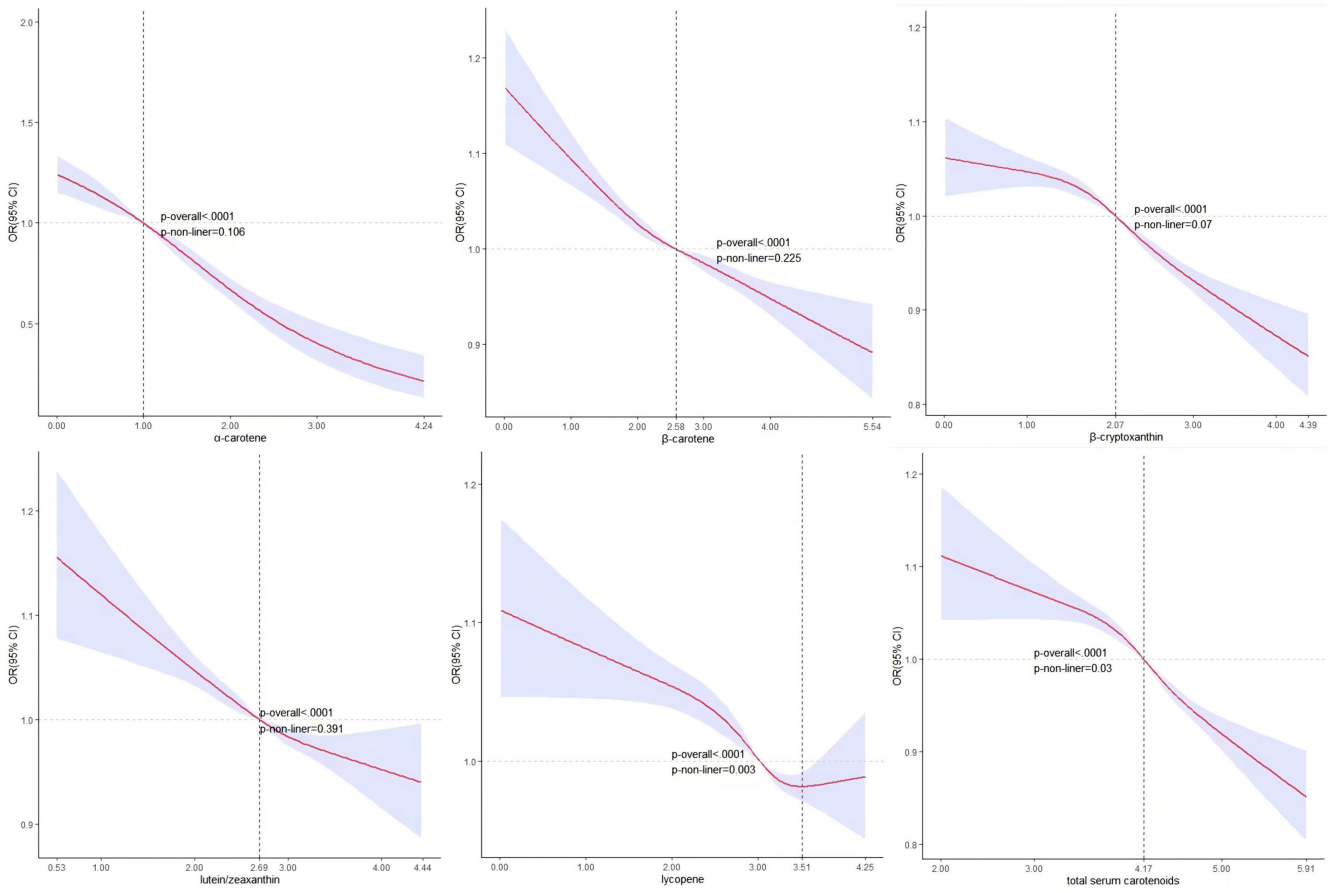
RCS analysis of serum carotenoid with hyperuricemia.

Figure 2 shows no significant nonlinear association between serum carotenoid, excluding lycopene, and hyperuricemia based on the RCS analysis. The corresponding OR = 1 values and serum concentrations were *α*-carotene (1.00, 2.71 μg/dL), *β*-carotene (2.58, 13.20 μg/dL), β-cryptoxanthin (2.07, 7.92 μg/dL), lutein/zeaxanthin (2.69, 14.73 μg/dL), and TSC (4.17, 64.72 μg/dL). Conversely, lycopene exhibited a statistically significant non-linear association with the likelihood of hyperuricemia (*p*-value for nonlinearity: <0.01), with an inflection point at 3.51 and a corresponding serum lycopene concentration of 33.45 μg/dL.

### 3.4 Stratified assessment

Table 7 evaluated how confounding factors and specific populations affect the relationship between serum carotenoids and hyperuricemia risk, while Table 8 focused on their impact on serum carotenoids and UA levels. The results showed that serum carotenoids were significantly associated with hyperuricemia in most population groups, except among Other Hispanic, Other Race, and individuals with a PIR of 1.0–3.0 (*p* > 0.05). Additionally, we examined how various patient characteristics might influence the observed associations. The analysis found significant interactions with age, race, education, alcohol status, PIR, BMI, hypertension, CVD, and diabetes (*P* for interaction<0.05), suggesting that these factors may modify the relationship between serum carotenoid and hyperuricemia.

**Table 7:** Subgroup analyses of the relationship between TSC and hyperuricemia.

TSC	Continuous	Q1	Q2	Q3	Q4	P for trend	P for interaction
Age							0.03
<60	0.61 (0.50, 0.73)	1	0.72 (0.58, 0.89)	0.65 (0.51, 0.82)	0.54 (0.41, 0.70)	<0.01	
≥60	0.73 (0.59, 0.89)	1	0.86 (0.64, 1.14)	0.75 (0.56, 1.01)	0.73 (0.53, 1.00)	<0.01	
Sex							0.71
Male	0.66 (0.55, 0.80)	1	0.84 (0.66, 1.06)	0.68 (0.53, 0.87)	0.62 (0.48, 0.81)	<0.01	
Female	0.60 (0.48, 0.74)	1	0.69 (0.52, 0.92)	0.67 (0.50, 0.90)	0.50 (0.36, 0.69)	<0.01	
Race							0.02
Non-Hispanic White	0.62 (0.52, 0.74)	1	0.74 (0.59, 0.92)	0.64 (0.51, 0.81)	0.56 (0.44, 0.72)	<0.01	
Non-Hispanic Black	0.64 (0.52, 0.78)	1	1.02 (0.78, 1.32)	0.64 (0.49, 0.85)	0.60 (0.45, 0.79)	<0.01	
Mexican American	0.61 (0.42, 0.87)	1	0.81 (0.51, 0.98)	0.75 (0.47, 0.86)	0.42 (0.24, 0.74)	<0.01	
Other Hispanic	0.49 (0.27, 0.88)	1	0.73 (0.35, 1.52)	0.39 (0.18, 0.87)	0.54 (0.24, 1.24)	<0.01	
Other Race	0.71 (0.46, 1.10)	1	1.18 (0.62, 2.23)	0.64 (0.32, 1.28)	0.66 (0.32, 1.36)	<0.01	
Education							<0.01
Below high school	0.61 (0.46, 0.80)	1	0.97 (0.64, 1.46)	0.46 (0.30, 0.71)	0.51 (0.33, 0.79)	<0.01	
High school	0.70 (0.54, 0.91)	1	0.79 (0.54, 1.15)	0.81 (0.56, 1.18)	0.65 (0.44, 0.96)	<0.01	
Above high school	0.48 (0.39, 0.60)	1	0.66 (0.51, 0.86)	0.51 (0.38, 0.67)	0.42 (0.31, 0.56)	<0.01	
Smoke							0.78
Current	0.69 (0.50, 0.95)	1	0.78 (0.52, 1.17)	0.72 (0.48, 1.08)	0.69 (0.44, 0.98)	<0.01	
Former	0.64 (0.50, 0.82)	1	0.78 (0.56, 1.08)	0.67 (0.48, 0.95)	0.70 (0.50, 0.99)	<0.01	
Never	0.61 (0.48, 0.76)	1	0.75 (0.58, 0.98)	0.64 (0.49, 0.84)	0.48 (0.35, 0.65)	<0.01	
Alcohol status, *n* (%)							<0.01
Non-drinker	0.64 (0.51, 0.80)	1	1.00 (0.72, 1.38)	0.61 (0.43, 0.86)	0.60 (0.40, 0.85)	<0.01	
1-5drinks/month	0.51 (0.43, 0.91)	1	0.69 (0.55, 0.87)	0.63 (0.50, 0.79)	0.44 (0.34, 0.56)	<0.01	
5-10drinks/month	0.61 (0.40, 0.91)	1	0.53 (0.30, 0.92)	0.78 (0.47, 1.31)	0.49 (0.28, 0.86)	<0.01	
>10 drinks/month	0.46 (0.37, 0.59)	1	0.87 (0.65, 1.25)	0.46 (0.32, 0.68)	0.37 (0.25, 0.54)	<0.01	
PIR, *n* (%)							<0.01
<1.0	0.58 (0.49, 0.70)	1	0.74 (0.59, 0.93)	0.70 (0.55, 0.89)	0.48 (0.37, 0.63)	<0.01	
1.0–3.0	0.81 (0.65, 1.00)	1	0.90 (0.68, 1.18)	0.71 (0.53, 0.95)	0.81 (0.60, 1.09)	<0.01	
>3.0	0.55 (0.45, 0.68)	1	0.75 (0.58, 0.97)	0.70 (0.53, 0.91)	0.44 (0.32, 0.60)	<0.01	
BMI							<0.01
<25	0.47 (0.37,0.65)	1	0.67 (0.45, 1.01)	0.60 (0.38, 0.94)	0.36 (0.22, 0.59)	<0.01	
≥25	0.62 (0.53, 0.72)	1	0.80 (0.66, 0.98)	0.62 (0.51, 0.76)	0.57 (0.46, 0.70)	<0.01	
Hypertension, *n* (%)							<0.01
1	0.78 (0.64, 0.96)	1	0.75(0.57,0.98)	0.74(0.56,0.98)	0.78(0.58,1.00)	0.3	
0	0.56 (0.46, 0.68)	1	0.76(0.56,0.95)	0.62(0.48,0.94)	0.47(0.35,0.62)	<0.01	
CVD, *n* (%)							<0.01
1	0.79 (0.57, 1.00)	1	0.70 (0.45, 1.10)	0.84 (0.52, 1.09)	0.56 (0.35, 0.89)	<0.01	
0	0.63 (0.53, 0.73)	1	0.80 (0.66, 0.96)	0.71 (0.58, 0.87)	0.61 (0.49, 0.76)	<0.01	
Diabetes, *n* (%)							<0.01
1	0.98 (0.68, 1.01)	1	0.76 (0.47, 1.23)	0.69 (0.43, 1.13)	0.58 (0.47, 0.98)	<0.01	
0	0.50 (0.50, 0.68)	1	0.76 (0.63, 0.96)	0.67 (0.55, 0.82)	0.55 (0.44, 0.68)	<0.01	

**Table 8:** Subgroup analyses of the relationship between TSC and SUA levels.

TSC	Continuous	Q1	Q2	Q3	Q4	P for trend	P for interaction
Age							0.30
<60	−0.15 (−0.23, −0.08)	1	−0.12 (−0.20, −0.03)	−0.17(−0.25, −0.08)	−0.21 (−0.31, −0.12)	<0.01	
≥60	−0.22 (−0.33, −0.12)	1	−0.05 (−0.22, 0.11)	−0.13 (−0.29, 0.03)	−0.25 (−0.41, −0.09)	<0.01	
Sex							0.12
Male	−0.18 (−0.26, −0.09)	1	−0.09(−0.21, 0.02)	−0.15 (−0.27, −0.04)	−0.22 (−0.34, −0.11)	<0.01	
Female	−0.17 (−0.26, −0.08)	1	−0.09 (−0.20, 0.10)	−0.16 (−0.26, −0.06)	−0.22 (−0.34, −0.11)	<0.01	
Race							0.02
Non-Hispanic White	−0.41 (−0.49, −0.34)	1	−0.16 (−0.26, −0.05)	−0.24 (−0.34, −0.14)	−0.55 (−0.65, −0.45)	<0.01	
Non-Hispanic Black	−0.32 (−0.45, −0.20)	1	−0.06 (−0.23, 0.11)	−0.31 (−0.48, −0.14)	−0.34 (−0.52, −0.17)	<0.01	
Mexican American	−0.59 (−0.71, −0.46)	1	−0.28 (−0.47, −0.09)	−0.36 (−0.54, −0.17)	−0.74 (−0.92, −0.56)	<0.01	
Other Hispanic	−0.35 (−0.64, −0.05)	1	−0.23 (−0.66, 0.19)	−0.34 (−0.78, 0.10)	−0.32 (−0.77, 0.14)	0.18	
Other Race	−0.45 (−0.72, −0.18)	1	−0.18 (−0.59, 0.22)	−0.39 (−0.77, 0.00)	−0.60 (−0.98, −0.23)	<0.01	
Education							0.01
Below high school	−0.55 (−0.71, −0.38)	1	−0.26 (−0.53, 0.02)	−0.44 (−0.70, −0.19)	−0.76 (−1.0, −0.50)	<0.01	
High school	−0.27 (−0.42, −0.12)	1	−0.03 (−0.23, 0.17)	−0.08 (−0.30, 0.13)	−0.39 (−0.61, −0.17)	<0.01	
Above high school	−0.42 (−0.56, −0.29)	1	−0.25 (−0.40, −0.10)	−0.37 (−0.52, −0.22)	−0.59 (−0.74, −0.44)	<0.01	
Smoke							0.01
Current	−0.22 (−0.36, −0.08)	1	−0.11 (−0.25, 0.04)	−0.14 (−0.31, 0.03)	−0.35 (−0.59, −0.12)	<0.01	
Former	−0.51 (−0.62, −0.39)	1	−0.23 (−0.40, −0.05)	−0.33 (−0.50, −0.16)	−0.56 (−0.73, −0.39)	<0.01	
Never	−0.52 (−0.61, −0.42)	1	−0.20 (−0.33, −0.06)	−0.39 (−0.51, −0.26)	−0.68 (−0.80, −0.56)	<0.01	
Alcohol status, *n* (%)							0.01
Non-drinker	−0.31 (−0.44, −0.18)	1	−0.03 (−0.22, 0.17)	−0.34 (−0.53, −0.15)	−0.40 (−0.59, −0.21)	<0.01	
1-5drinks/month	−0.42 (−0.51, −0.34)	1	−0.20 (−0.32, −0.07)	−0.28 (−0.40, −0.15)	−0.54 (−0.66, −0.41)	<0.01	
5-10drinks/month	−0.33 (−0.72, 0.05)	1	−0.28 (−0.60, 0.04)	−0.12 (−0.45, 0.20)	−0.59 (−0.92, −0.27)	<0.01	
>10 drinks/month	−0.54 (−0.67, −0.42)	1	−0.14 (−0.36, 0.08)	−0.41 (−0.63, −0.19)	−0.68 (−0.90, −0.46)	<0.01	
PIR, *n* (%)							0.3
<1.0	−0.11 (−0.26, −0.04)	1	−0.20 (−0.39, −0.01)	−0.22 (−0.43, −0.01)	−0.45 (−0.63, −0.27)	<0.01	
1.0–3.0	−0.12 (−0.21, −0.02)	1	−0.16(−0.30, −0.03)	−0.30 (−0.43, −0.17)	−0.51 (−0.64, −0.37)	<0.01	
>3.0	−0.18 (−0.28, −0.08)	1	−0.20 (−0.33, −0.06)	−0.33 (−0.47, −0.20)	−0.65 (−0.78, −0.51)	<0.01	
BMI							0.11
<25	−0.17 (−0.26, −0.08)	1	−0.10 (−0.23, 0.03)	−0.19 (−0.32, −0.05)	−0.22 (−0.35, −0.10)	<0.01	
≥25	−0.25 (−0.34, −0.16)	1	−0.14 (−0.24, −0.05)	−0.20 (−0.30, −0.11)	−0.33 (−0.44, −0.23)	<0.01	
Hypertension, *n* (%)							0.21
1	−0.14 (−0.26, −0.03)	1	−0.16 (−0.31, −0.01)	−0.11 (−0.26, −0.04)	−0.16 (−0.33, −0.01)	0.14	
0	−0.25 (−0.34, −0.16)	1	−0.08 (−0.17, 0.01)	−0.16 (−0.25, −0.07)	−0.23 (−0.32, −0.14)	<0.01	
CVD, *n* (%)							0.42
1	−0.06 (−0.29, 0.18)	1	0.05 (−0.23, 0.33)	−0.01 (−0.27, 0.29)	−0.10 (−0.42, 0.22)	<0.01	
0	−0.17 (−0.24, −0.01)	1	−0.12 (−0.20, −0.04)	−0.17 (−0.25, −0.09)	−0.23 (−0.31, −0.15)	<0.01	
Diabetes, *n* (%)							0.08
1	−0.02 (−0.23, 0.19)	1	0.14 (−0.40, 0.12)	−0.16 (−0.44, 0.11)	−0.13 (−0.20, 0.46)	0.23	
0	−0.19 (−0.26, −0.12)	1	−0.11 (−0.19, −0.03)	−0.16 (−0.24, −0.08)	−0.26 (−0.34, −0.17)	<0.01	

## 4 Discussion

This population-based study found that higher serum levels of carotenoids, including *α*-carotene, *β*-carotene, β-cryptoxanthin, lutein/zeaxanthin, and TSC, are associated with lower UA levels. However, lycopene showed no significant correlation with UA levels. Higher serum carotenoid levels were significantly associated with a decreased incidence of hyperuricemia. RCS analysis indicates no significant nonlinear association between α-carotene, *β*-carotene, β-cryptoxanthin, lutein/zeaxanthin, or TSC and hyperuricemia. In contrast, a potential nonlinear relationship was observed between lycopene and hyperuricemia, with an inflection point at 33.45 μg/dL. Subgroup analyses and interaction tests suggest that this positive correlation is consistent across different demographic groups.

Our findings are consistent with previous studies. An analysis of data from 10,893 adults in a national survey conducted from 2001 to 2006 in the United States found that *β*-carotene concentrations were inversely associated with UA levels (22). Another study using data from 14,349 participants in the third NHANES (1988–1994) found that β-carotene intake may help protect against hyperuricemia (23). However, our study is more comprehensive than previous research, as it examines the relationships between six major serum carotenoids and UA levels as well as hyperuricemia.

Oxidative stress, defined as an imbalance between reactive oxygen species (ROS) and the body’s antioxidant defenses, plays a key role in many chronic diseases, including hyperuricemia (24). Prolonged oxidative stress can damage critical tissues and cells, particularly in organs involved in UA metabolism, such as the liver and kidneys (25, 26). Carotenoids, a diverse group of phytochemicals synthesized by plants and microorganisms, exhibit strong singlet molecular oxygen quenching and free radical-scavenging activities, thereby protecting biological systems from oxidative damage (27, 28). It has been reported that lycopene can directly react with free radicals to form more stable products, interrupting the chain reaction of free radicals, preventing the initiation of lipid peroxidation, reducing oxidative damage to cell membranes, and protecting proteins and DNA from oxidative stress (29). Additionally, carotenoids inhibit xanthine oxidase, a key enzyme in UA production, thus reducing UA levels (30, 31).

Moreover, carotenoids possess significant anti-inflammatory properties. By inhibiting key inflammatory mediators, such as NF-κB and cytokines like TNF-*α* and IL-6, carotenoids can reduce systemic inflammation, which may indirectly contribute to lowering UA levels (32–34). Chronic inflammation has been linked to hyperuricemia, as demonstrated by cross-sectional studies showing a strong correlation between dietary inflammation and elevated UA levels (5).

Another key mechanism involves carotenoids’ role in modulating renal UA transport proteins. Carotenoids may influence the expression and function of urate transporters, such as URAT1, GLUT9, and OAT4, which are essential for UA reabsorption and excretion in the kidneys (35). By promoting the excretion of UA through these pathways, carotenoids can help maintain lower UA levels. Lutein and zeaxanthin also contribute to renal protection by preserving renal function, crucial for UA clearance (36, 37). Their combined antioxidant and anti-inflammatory actions protect renal tissues from damage, particularly in individuals with chronic kidney disease or impaired renal function, both of which are strongly associated with hyperuricemia (38).

Beyond these mechanisms, individual carotenoids may also impact metabolic pathways associated with hyperuricemia. For instance, *β*-carotene and lutein have been shown to improve insulin sensitivity, a key factor in enhancing UA excretion, as insulin resistance is known to impair this process (39, 40). Carotenoids may promote weight loss, lower blood glucose levels, and reduce blood lipid levels (41–43). These combined effects improve overall metabolic health, thereby reducing the risk of developing hyperuricemia. Additionally, emerging research suggests that *β*-carotene and *α*-carotene could influence gut microbiota composition, reducing intestinal absorption of purines and enhancing microbial degradation of UA, further contributing to the reduction of UA levels (33).

Most carotenoids, such as β-carotene and lutein/zeaxanthin, show a linear negative correlation with hyperuricemia. However, lycopene exhibits a different trend, with RCS analysis revealing a more complex, non-linear relationship between lycopene and hyperuricemia. This may be related to the unique bioactivity and metabolic characteristics of lycopene. Lycopene has a very strong antioxidant capacity, especially in quenching singlet oxygen. However, its unique structure (11 conjugated double bonds) makes its reactivity different from other carotenoids (44). Lycopene tends to accumulate in adipose tissue and the liver, which may affect its specific role in UA metabolism (45). In contrast, *β*-carotene, lutein, and other carotenoids are more widely distributed in different tissues and reduce serum UA levels through various mechanisms, including stronger anti-inflammatory effects and renal protection. Therefore, while lycopene is highly effective as an antioxidant, its ability to inhibit UA production and promote excretion may not be as consistent as other carotenoids. Future research should further explore the effects of lycopene and other carotenoids on UA metabolism under different doses and conditions.

Subgroup analysis further revealed that factors such as age, gender, and race modulate the relationship between carotenoids and UA or hyperuricemia. For example, the negative correlation between TSC and hyperuricemia is more pronounced in participants under 60 years old than in older individuals, suggesting that younger people may gain more protective effects from carotenoids. Regarding gender, the negative correlation between carotenoid levels and hyperuricemia is slightly stronger in women than in men, which may be related to women’s stronger carotenoid metabolism and antioxidant capacity (46). Race is also an important moderating factor. Subgroup analysis shows that the negative correlation between TSC and hyperuricemia is most significant among Mexican Americans, possibly due to their diet being richer in carotenoid-containing foods. Table 2 also indicates that participants with higher TSC levels tend to have higher education and income levels. These socioeconomic factors may promote an increase in carotenoid levels through healthier lifestyles, such as higher fruit and vegetable intake and reduced smoking and alcohol consumption. Subgroup analysis supports this view, as the negative correlation between TSC and hyperuricemia is more significant among individuals with higher education and income levels. Smoking appears to weaken the protective effects of carotenoids, as the negative correlation between TSC and hyperuricemia is less pronounced in smokers, likely due to increased oxidative stress that reduces carotenoids’ antioxidant capacity. In contrast, the relationship between TSC and hyperuricemia is stronger in drinkers, particularly heavy drinkers. This unexpected finding could be explained by the higher baseline prevalence of hyperuricemia in drinkers, as alcohol metabolism increases lactate levels, which compete with UA for excretion, leading to elevated serum UA levels. This higher UA level may provide more opportunity for carotenoids to exert their protective effects. The antioxidant and anti-inflammatory properties of carotenoids may help mitigate the oxidative stress and inflammation caused by alcohol, resulting in a stronger protective effect in this group. These findings highlight the complex interactions between carotenoids and lifestyle factors like smoking and alcohol consumption, warranting further research to understand how alcohol intake influences the health benefits of carotenoids, particularly in populations at risk for hyperuricemia.

The major strengths of this study are as follows. First, it is the first study to use a nationally representative sample to examine the association between serum carotenoids, UA, and hyperuricemia. Second, we adjusted for a broad range of potential confounding variables. Third, the accuracy and reliability of the data were enhanced by using trained staff who followed standardized protocols to assess the main information and conduct interviews with study participants. Our study also has some limitations. First, as a cross-sectional study, this study cannot establish causal relationships. Therefore, further prospective longitudinal studies are needed to confirm these findings. Second, due to limitations in data collection, we were unable to include other dietary factors related to UA levels, such as seafood and beer, in the adjustment model. Third, measuring serum carotenoids at a single point in time may not accurately reflect the effects of continuous exposure.

## 5 Conclusion

This study revealed a significant inverse relationship between serum carotenoids, except lycopene, and UA levels. Higher levels of serum carotenoids were significantly associated with a decreased incidence of hyperuricemia. For the general population, increased serum carotenoid levels may lower the risk of hyperuricemia, which could be valuable in developing preventive strategies.

## Data Availability

The original contributions presented in the study are included in the article/supplementary material, further inquiries can be directed to the corresponding authors.

## References

[1] Valsaraj R Singh AK Gangopadhyay KK Ghoshdastidar B Goyal G Batin M. Management of asymptomatic hyperuricemia: Integrated Diabetes & Endocrine Academy (IDEA) consensus statement. Diabetes Metab Syndr. (2020) 14:93–100. doi: 10.1016/j.dsx.2020.01.007, PMID: 31991299

[2] Major TJ Dalbeth N Stahl EA Merriman TR. An update on the genetics of hyperuricaemia and gout. Nat Rev Rheumatol. (2018) 14:341–53. doi: 10.1038/s41584-018-0004-x, PMID: 29740155

[3] Dehlin M Jacobsson L Roddy E. Global epidemiology of gout: prevalence, incidence, treatment patterns and risk factors. Nat Rev Rheumatol. (2020) 16:380–90. doi: 10.1038/s41584-020-0441-1, PMID: 32541923

[4] Chen-Xu M Yokose C Rai SK Pillinger MH Choi HK. Contemporary prevalence of gout and hyperuricemia in the United States and decadal trends: the National Health and nutrition examination survey, 2007-2016. Arthritis Rheumatol. (2019) 71:991–9. doi: 10.1002/art.40807, PMID: 30618180 PMC6536335

[5] Wang H Qin S Li F Zhang H Zeng L. A cross-sectional study on the association between dietary inflammatory index and hyperuricemia based on NHANES 2005-2018. Front Nutr. (2023) 10:1218166. doi: 10.3389/fnut.2023.1218166, PMID: 37810924 PMC10552180

[6] Danve A Sehra ST Neogi T. Role of diet in hyperuricemia and gout. Best Pract Res Clin Rheumatol. (2021) 35:101723. doi: 10.1016/j.berh.2021.101723, PMID: 34802900 PMC8678356

[7] Teramura S Yamagishi K Umesawa M Hayama-Terada M Muraki I Maruyama K. Risk factors for hyperuricemia or gout in men and women: the circulatory risk in communities study (CIRCS). J Atheroscler Thromb. (2023) 30:1483–91. doi: 10.5551/jat.63907, PMID: 36878531 PMC10564651

[8] Fang XY Qi LW Chen HF Gao P Zhang Q Leng RX. The interaction between dietary fructose and gut microbiota in hyperuricemia and gout. Front Nutr. (2022) 9:890730. doi: 10.3389/fnut.2022.890730, PMID: 35811965 PMC9257186

[9] Gu T Cao G Luo M Zhang N Xue T Hou R. A systematic review and meta-analysis of the hyperuricemia risk from certain metals. Clin Rheumatol. (2022) 41:3641–60. doi: 10.1007/s10067-022-06362-1, PMID: 36109472

[10] Li Z Gao L Zhong X Feng G Huang F Xia S. Association of Visceral fat Area and Hyperuricemia in non-obese US adults: a cross-sectional study. Nutrients. (2022) 14:3992. doi: 10.3390/nu14193992, PMID: 36235645 PMC9570922

[11] Vargas-Santos AB Neogi T. Management of Gout and Hyperuricemia in CKD. Am J Kidney Dis. (2017) 70:422–39. doi: 10.1053/j.ajkd.2017.01.055, PMID: 28456346 PMC5572666

[12] Cheng S Shan L You Z Xia Y Zhao Y Zhang H. Dietary patterns, UA levels, and hyperuricemia: a systematic review and meta-analysis. Food Funct. (2023) 14:7853–68. doi: 10.1039/D3FO02004E, PMID: 37599588

[13] Terao J. Revisiting carotenoids as dietary antioxidants for human health and disease prevention. Food Funct. (2023) 14:7799–824. doi: 10.1039/D3FO02330C, PMID: 37593767

[14] Eroglu A Al'Abri IS Kopec RE Crook N Bohn T. Carotenoids and their health benefits as derived via their interactions with gut microbiota. Adv Nutr. (2023) 14:238–55. doi: 10.1016/j.advnut.2022.10.007, PMID: 36775788 PMC10229386

[15] Yabuzaki J. Carotenoids database: structures, chemical fingerprints and distribution among organisms. Database. (2017) 2017:bax004. doi: 10.1093/database/bax004, PMID: 28365725 PMC5574413

[16] Kurniawan R Nurkolis F Taslim NA Subali D Surya R Gunawan WB. Carotenoids composition of green algae *Caulerpa racemosa* and their antidiabetic, anti-obesity, antioxidant, and anti-inflammatory properties. Molecules. (2023) 28:3267. doi: 10.3390/molecules28073267, PMID: 37050034 PMC10096636

[17] Aguilar SS Wengreen HJ Lefevre M Madden GJ Gast J. Skin carotenoids: a biomarker of fruit and vegetable intake in children. J Acad Nutr Diet. (2014) 114:1174–80. doi: 10.1016/j.jand.2014.04.026, PMID: 24951435

[18] Ahluwalia N Dwyer J Terry A Moshfegh A Johnson C. Update on NHANES dietary data: focus on collection, release, analytical considerations, and uses to inform public policy. Adv. Nutr. (2016) 7:121–34. doi: 10.3945/an.115.009258, PMID: 26773020 PMC4717880

[19] Zou Q Wang H Su C Du W Ouyang Y Jia X. Longitudinal association between physical activity and blood pressure, risk of hypertension among Chinese adults: China health and nutrition survey 1991–2015. Eur J Clin Nutr. (2021) 75:274–82. doi: 10.1038/s41430-020-0653-0, PMID: 32404900

[20] Csete J Kamarulzaman A Kazatchkine M Altice F Balicki M Buxton J. Public health and international drug policy. Lancet. (2016) 387:1427–80. doi: 10.1016/S0140-6736(16)00619-X, PMID: 27021149 PMC5042332

[21] Zhu X Cheang I Tang Y Shi M Zhu Q Gao R. Associations of serum carotenoids with risk of all-cause and cardiovascular mortality in hypertensive adults. J Am Heart Assoc. (2023) 12:e027568. doi: 10.1161/JAHA.122.027568 36752230 PMC10111495

[22] Ford ES Choi HK. Associations between concentrations of UA with concentrations of vitamin a and beta-carotene among adults in the United States. Nutr Res. (2013) 33:995–1002. doi: 10.1016/j.nutres.2013.08.008, PMID: 24267038 PMC4589134

[23] Choi WJ Ford ES Curhan G Rankin JI Choi HK. Independent association of serumretinol and β-carotene levels with hyperuricemia: a national population study. Arthritis Care Res. (2012) 64:389–96. doi: 10.1002/acr.20692 PMC3288639 22076806

[24] Liu Y Gong S Li K Wu G Zheng X Zheng J. Coptisine protects against hyperuricemic nephropathy through alleviating inflammation, oxidative stress and mitochondrial apoptosis via PI3K/Akt signaling pathway. Biomed Pharmacother. (2022) 156:113941. doi: 10.1016/j.biopha.2022.113941 36411660

[25] Gherghina ME Peride I Tiglis M Neagu TP Niculae A Checherita IA. UA and oxidative stress-relationship with cardiovascular, metabolic, and renal impairment. Int J Mol Sci. (2022) 23:3188. doi: 10.3390/ijms23063188, PMID: 35328614 PMC8949471

[26] Krishnan E. Inflammation, oxidative stress and lipids: the risk triad for atherosclerosis in gout. Rheumatology. (2010) 49:1229–38. doi: 10.1093/rheumatology/keq037 20202928

[27] Gammone MA Riccioni G D'Orazio N. Marine carotenoids against oxidative stress: effects on human health. Mar Drugs. (2015) 13:6226–46. doi: 10.3390/md13106226, PMID: 26437420 PMC4626686

[28] Manochkumar J Doss CGP El-Seedi HR Efferth T Ramamoorthy S. The neuroprotective potential of carotenoids in vitro and in vivo. Phytomedicine. (2021) 91:153676. doi: 10.1016/j.phymed.2021.153676, PMID: 34339943

[29] Przybylska S Tokarczyk G. Lycopene in the prevention of cardiovascular diseases. Int J Mol Sci. (2022) 23:1957. doi: 10.3390/ijms23041957, PMID: 35216071 PMC8880080

[30] Liu CC Huang CC Lin WT Hsieh CC Huang SY Lin SJ. Lycopene supplementation attenuated xanthine oxidase and myeloperoxidase activities in skeletal muscle tissues of rats after exhaustive exercise. Br JNutr. (2005) 94:595–601. doi: 10.1079/BJN20051541 16197586

[31] Kozuki Y Miura Y Yagasaki K. Inhibitory effects of carotenoids on the invasion of rat ascites hepatoma cells in culture. Cancer Lett. (2000) 151:111–5. doi: 10.1016/S0304-3835(99)00418-8 10766430

[32] Milani A Basirnejad M Shahbazi S Bolhassani A. Carotenoids: biochemistry, pharmacology and treatment. Br J Pharmacol. (2017) 174:1290–324. doi: 10.1111/bph.13625, PMID: 27638711 PMC5429337

[33] Rocha HR Coelho MC Gomes AM Pintado ME. Carotenoids diet: digestion, gut microbiota modulation, and inflammatory diseases. Nutrients. (2023) 15:2265. doi: 10.3390/nu15102265, PMID: 37242148 PMC10220829

[34] Ciccone MM Cortese F GeUAldo M Carbonara S Zito A Ricci G. Dietary intake of carotenoids and their antioxidant and anti-inflammatory effects in cardiovascular care. Mediat Inflamm. (2013) 2013:782137:1–11. doi: 10.1155/2013/782137 PMC3893834 24489447

[35] Le Y Zhou X Zheng J Yu F Tang Y Yang Z. Anti-Hyperuricemic effects of Astaxanthin by regulating xanthine oxidase, adenosine deaminase and urate transporters in rats. Mar Drugs. (2020) 18:610. doi: 10.3390/md18120610, PMID: 33271765 PMC7759838

[36] Hirahatake KM Jacobs DR Gross MD Bibbins-Domingo KB Shlipak MG Mattix-Kramer H. The Association of Serum Carotenoids, tocopherols, and ascorbic acid with rapid kidney function decline: the coronary artery risk development in young adults (CARDIA) study. J Ren Nutr. (2019) 29:65–73. doi: 10.1053/j.jrn.2018.05.008, PMID: 30098859

[37] Bedir F Kocaturk H Turangezli O Sener E Akyuz S Ozgeris FB. The protective effect of lycopene against oxidative kidney damage associated with combined use of isoniazid and rifampicin in rats. Braz J Med Biol Res. (2021) 54:e10660. doi: 10.1590/1414-431x2020e10660, PMID: 34037090 PMC8148980

[38] Samarghandian S Azimi-Nezhad M Borji A Farkhondeh T. Effect of crocin on aged rat kidney through inhibition of oxidative stress and proinflammatory state. Phytother Res. (2016) 30:1345–53. doi: 10.1002/ptr.5638, PMID: 27279282

[39] Borghi C Agabiti-Rosei E Johnson RJ Kielstein JT Lurbe E Mancia G. Hyperuricaemia and gout in cardiovascular, metabolic and kidney disease. Eur J Intern Med. (2020) 80:1–11. doi: 10.1016/j.ejim.2020.07.006 32739239

[40] Mummidi S Farook VS Reddivari L Hernandez-Ruiz J Diaz-Badillo A Fowler SP. Serum carotenoids and pediatric metabolic index predict insulin sensitivity in Mexican American children. Sci Rep. (2021) 11:871. doi: 10.1038/s41598-020-79387-8, PMID: 33441626 PMC7806924

[41] Bonet ML Canas JA Ribot J Palou A. Carotenoids and their conversion products in the control of adipocyte function, adiposity and obesity. Arch Biochem Biophys. (2015) 572:112–25. doi: 10.1016/j.abb.2015.02.022 25721497

[42] Evangelista-Silva PH Prates RP Leite JSM Moreno LG Goulart-Silva F Esteves EA. Intestinal GLUT5 and FAT/CD36 transporters and blood glucose are reduced by a carotenoid/MUFA-rich oil in high-fat fed mice. Life Sci. (2021) 279:119672. doi: 10.1016/j.lfs.2021.119672, PMID: 34097971

[43] Morris DL Kritchevsky SB Davis CE. Serum carotenoids and coronary heart disease. The lipid research clinics coronary primary prevention trial and follow-up study. JAMA. (1994) 272:1439–41. doi: 10.1001/jama.1994.03520180063036 7933426

[44] Singh J Jayaprakasha GK Patil BS. Improved sample preparation and optimized solvent extraction for quantitation of carotenoids. Plant Foods Hum Nutr. (2021) 76:60–7. doi: 10.1007/s11130-020-00862-8, PMID: 33420704

[45] Baz L Algarni S Al-Thepyani M Aldairi A Gashlan H. Lycopene improves metabolic disorders and liver injury induced by a Hight-fat diet in obese rats. Molecules. (2022) 27:7736. doi: 10.3390/molecules27227736, PMID: 36431836 PMC9699056

[46] Allore T Lemieux S Vohl MC Couture P Lamarche B Couillard C. Correlates of the difference in plasma carotenoid concentrations between men and women. Br J Nutr. (2019) 121:172–81. doi: 10.1017/S0007114518003045, PMID: 30392471

